# Five years after the accident, whiplash casualties still have poorer quality of life in the physical domain than other mildly injured casualties: analysis of the ESPARR cohort

**DOI:** 10.1186/s12889-015-2647-8

**Published:** 2016-01-05

**Authors:** Charlène Tournier, Martine Hours, Pierrette Charnay, Laetitia Chossegros, Hélène Tardy

**Affiliations:** Unité Mixte de Recherche Epidémiologique et de Surveillance Transport Travail Environnement (UMRESTTE UMR_T 9405), Université Lyon 1, Université de Lyon, Lyon, France

**Keywords:** Whiplash, Quality of life, Comparison, ESPARR Cohort, Prospective study, 5 year follow-up, Mild injury, Road-accident injury

## Abstract

**Background:**

This study aims to compare health status and quality of life five years after a road accident between casualties with whiplash versus other mild injuries, to compare evolution of quality of life at 1 and 5 years after the accident, and to explore the relation between initial injury (whiplash vs. other) and quality of life.

**Methods:**

The study used data from the ESPARR cohort (a representative cohort of road accident casualties) and included 167 casualties with “pure” whiplash and a population of 185 casualties with other mild injuries (MAIS-1). All subjects with lesions classified as cervical contusion (AIS code 310402) or neck sprain (AIS code 640278) were considered as whiplash casualties. Diagnosis was made by physicians, at the outset of hospital care, based on interview, clinical findings and X-ray. Whiplash injuries were then classified following the Quebec classification (grades 1 and 2). Quality of life was assessed on the WHOQoL-Bref questionnaire. Correlations between explanatory variables and quality of life were explored by Poisson regression and variance analysis.

**Results:**

Between 1 and 5 years, global QoL improved for both whiplash and non-whiplash casualties; but, considering the two whiplash groups separately, improvement in grade 2 was much less than in grade 1. At 5 years, grade-2 whiplash casualties were more dissatisfied with their health (39.4 %; *p* < 0.05) than non-whiplash (24.3 %) or grade-1 whiplash casualties (27.0 %). Deteriorated quality of life in the mental, social and environmental domains was mainly related to psychological and socioeconomic factors for both whiplash and other mildly injured road-accident casualties. While PTSD was a major factor for the physical domain, whiplash remained a predictive factor after adjustment on PTSD; unsatisfactory health at 5 years, with deteriorated quality of life in the physical domain, was observed specifically in the whiplash group, pain playing a predominant intermediate role.

**Conclusions:**

Deteriorated quality of life in the physical domain remained 5 years after the accident, specifically in the grade-2 whiplash group, pain playing a predominant intermediate role, which may be in line with the hypothesis of neuropathic pain.

## Background

Defined as an acceleration-deceleration mechanism in the neck, whiplash is the most common injury in road accidents, particularly for motorists: over the 2007–2010 period (French road safety database), 35 % of injured car occupants were concerned [1]. The annual incidence rate of casualties suffering from whiplash seems to be of the same order of magnitude in most countries (annual incidence between 0.04 and 3.2/1,000 inhabitants [2, 3]). A study of drivers in collisions involving two cars found similar results in French (1997–2003) and Spanish databases (2002–2004) [4]: 12.2 % were diagnosed with whiplash in France and 12.0 % in Spain.

Although considered a minor injury, whiplash is reported to generate both short- and long-term consequences, such as neck pain, headache, dizziness, sensory disorder and reduced neck mobility. These symptoms are often grouped together as “whiplash-associated disorder” (WAD) [5]. Whiplash injury and WAD have been widely described in the international literature: in most studies, more than half of whiplash casualties reported non-recovery 1 year after the accident [6–11]. Many studies also examined the relation between quality of life (QoL) and whiplash [8, 12, 13], some showing a correlation between post-traumatic stress disorder and lower QoL [14]. Psychological factors, such as psychological history …., are often related to WAD [15, 16]. However, it is not clear if psychological factors are necessary and sufficient for non-recovery or if other sociodemographic or genetic factors may also play some role.

However, only a few studies [17, 18] compared chronicity in whiplash casualties versus other types of injury of similar severity. Chronic symptomatology may not be specific to whiplash, but dependent on personal (e.g., psychological) factors, as reported by the authors. If, however, whiplash casualties (unlike those with other comparable injuries) suffer specifically from chronical symptoms, and notably pain, it is necessary to further explore the possibility of neural lesions in the cervical region which may be the cause of neuropathic pain, as studied by Sterling and Pedler [19]. Furthermore, long-term (>1-year) follow-up of whiplash casualties is little documented in the literature [20–22].

A previous analysis of the mildly injured subjects of the ESPARR road-accident casualties cohort (ESPARR: *Étude et Suivi d*’*une Population d*’*Accidentés de la Route dans le Rhône*) showed that, 1 year after a low-severity road accident, subjects suffering from whiplash had recovered poorly compared to subjects with other minor injuries, that but QoL did not differ between whiplash and non-whiplash casualties [23]. The present study aimed to study the consequences of whiplash injury in the ESPARR cohort 5 years after the accident, in terms of pain, sequelae and QoL, and to compare them with those observed in the other mildly injured subjects. A secondary objective was to look for factors (notably whiplash status) associated with poorer quality of life in mildly injured subjects 5 years after the accident. Two underlying hypotheses were that psychological factors are significantly associated with persistent complaints, whatever the type of mild injury (not only whiplash) and that, in WAD, persistent pain accounts for complaints even in the absence of contributory psychological factors.

## Materials and methods

### The ESPARR cohort

The ESPARR project is a prospective cohort study of road accident casualties, which seeks to identify long-term consequences and to better define what constitutes serious injury. It is based on the Rhône Registry of Road Traffic Casualties [24], which, since 1995, has recorded all casualties receiving medical care in public or private health facilities in the Rhône administrative area of France. The inclusion criteria are: (1) having had a road traffic accident in the Rhône administrative area involving at least one mechanical means of transport; (2) living in the Rhône administrative area; (3) having survived the accident at least up to hospital admission; and (4) having received care in one of the area’s hospitals. The injured are followed up for 5 years after the accident. All subjects have provided written consent.

Each individual lesion is coded using the Abbreviated Injury Scale (AIS) [25]: from 1 (minor injury) to 6 (fatal injury). The Maximum-AIS (M.AIS) is the injury’s highest AIS score and defines overall initial injury severity.

The inclusion period for road accidents was initially from October 1^st^, 2004 to December 31^st^, 2005, later extended to July 31^st^, 2006 for the most seriously injured (M.AIS ≥ 3). The protocol has been described in detail in a previous publication [26].

The cohort comprises 1,168 adults, aged ≥16 years at the time of the accident. At inclusion, the injured were asked to answer a questionnaire specifically drawn up for the ESPARR study, administered in a face-to-face interview.

The questionnaire gathered data on the accident and previous familial, occupational and health status. In addition, initial lesion assessment and other medical data (radiology, intensive care, etc.) were collected from medical records. Lesions were coded by the trained physician of the Registry from which the cohort was derived.

### Study population

The present study selected the 546 adults in the ESPARR cohort who had sustained only mild injury, defined as maximum AIS level 1 (MAIS-1), excluding cases with associated AIS ≥ 2 lesions in other body regions; 253 of these subjects had sustained whiplash injury and the other 293 had any other type of MAIS-1 lesion, such as ankle or shoulder sprain, superficial wounds or contusions, tendon tear, etc. In all, 352 subjects (64.5 %) responded to the 5-year follow-up questionnaire (between October 2009 and December 2010): 167 of the 253 whiplash cases (66.0 %), comprising 63 grade-1 and 104 grade-2 injuries, and 185 of the 293 other mild injury casualties (63.1 %). 16 % of non-whiplash and 20 % of whiplash casualties failed to respond to the 1-year questionnaire.

### Clinical definition of whiplash

In the present study, all subjects with lesions classified as cervical contusion (AIS code 310402) or neck sprain (AIS code 640278) were considered as whiplash casualties. Diagnosis was made by physicians, at the outset of hospital care, based on interview, clinical findings and X-ray. In the AIS classification, Code 310402 is attributed to neck pain following a road accident with painful neck on palpation without other objective signs, and corresponds to grade 1 in the Quebec classification; Code 640278 is attributed to neck pain associated with cervical stiffness and radiologic loss of cervical lordosis, and corresponds to grade 2 in the Quebec classification [23]. Cervical spine lesions graded AIS-1 (code 640278) but with associated neurologic abnormality (Quebec grade 3) (*n* = 2) were excluded.

### Variables and measurements

#### Outcome measurements at 5 years

QoL was evaluated on the World Health Organization Quality of Life (Brief) (WHOQoL-Bref) questionnaire [27]. This tool contains 26 questions. The first two assess perception of overall QoL and perception of overall health, respectively. The other questions are categorized in four domains: physical, psychological, social and environmental [28]. Responses to each question are graded on a five-point Likert scale for intensity, capacity, frequency or evaluation, as the case may be (from 1 = poor QoL, to 5 = good QoL). Each of the four scores (one per domain) ranges from 4 to 20, modified in the present study to 0–100 for comparison with WHOQOL-100 scores used in other studies. A high score indicates good QoL.

For analysis, the two generic questions (overall QoL and general health status) of the WHOQoL-Bref were coded as dichotomous variables: good or very good versus neither good nor bad, bad, very bad or no reply for overall QoL; and satisfied or very satisfied versus neither satisfied nor dissatisfied, dissatisfied, very dissatisfied or no reply for perception of overall health.

#### Variable of interest

The variable of interest for analysis was whiplash status, in three categories: non-whiplash, grade-1 whiplash, and grade-2 whiplash.

#### Exploratory variables

At inclusion, the questionnaire collected sociodemographic and accident-related data, plus some medical information and psychological history. Specifically, sociodemographic data comprised age, gender, family situation, educational level and socio-occupational category. Accident-related information comprised type of road-user with position inside the vehicle and impact direction, antagonist, reason for travel at time of accident, subjective responsibility for accident (admitting being at fault in the accident or not), friend or family member also injured in the same accident, and intention to lodge a complaint^1^. Financial problems (job loss, financial difficulties, failure) in the year before the accident and psychological history (sleep disorder, consumption of antidepressants/anxiolytics, regular appointments with a psychologist) in the year before the accident were also considered in this analysis.

Post-traumatic stress disorder (PTSD) at 6 months and/or 1 year after the accident was also taken into account and was assessed using the Post-traumatic CheckList Scale (PCLS) [29]. The PCLS includes 17 items relating to the 3 dimensions of the disorder (re-experiencing, avoidance and hyper-arousal), and has been shown to have good specificity for the diagnosis of PTSD; validation studies of the PCLS [29–32] have shown good psychometric properties. The validation of the French version [31] showed that PCLS score ≥ 44 indicates presence of PTSD and the existence of disturbances which will necessarily affect lifestyle.

The presence of pain and sequelae of accident-related injuries was collected in the 5-year questionnaire. A free text about pain location allowed data to be classified according to body region (head, neck, face, spine, thorax, abdomen, upper limbs, and lower limbs); a dichotomous variable was created for each region (pain vs. no pain).

### Statistical analysis

The representativeness of the study population (whiplash and non-whiplash subjects) was assessed by comparing sociodemographic and accident data between respondents and non-respondents at 5 years’ follow-up. Descriptive statistics were used to describe the distribution of variables. Chi^2^ test (significance level, 5 %) or Fisher’s exact test (small samples) were used for categoric variables; Student’s test (normal data distribution) or Kruskal-Wallis test (non-normal data distribution) were used for continuous variables.

QoL data (the two generic questions and mean scores in the 4 domains) collected at 1 and 5 years after the accident were compared for subjects responding at the both follow-up steps. Thus, it was possible to detect a significant improvement or deterioration in QoL for each group, using McNemar’s test (significance level, 5 %) or Student’s test for matched data.

The next step was to identify factors for impaired QoL, with whiplash status as the variable of interest. Two modified Poisson regression models were built for each of the first two variables of the QoL scale: i.e., poor overall QoL and unsatisfactory overall health. Variance analysis was used to study each of the four QoL domains. Analysis strategy was identical in all models. Age and gender, considered as adjustment variables, were included in the multivariate analysis regardless of their significance level. The variable of interest (non-whiplash, grade-1 whiplash, grade-2 whiplash) and explanatory factors at time of accident or at 1-year follow-up significantly associated (≥10 %) with outcome on univariate analysis were included in the multivariate analysis, after checking for collinearity between explanatory variables. Stepwise selection with backward elimination was applied, with *p* > 0.05 for exclusion (model 1). For each outcome, the final model was built from model 1, to which the variable “pain at 5 years” was added (model 2).

QoL was also analyzed separately for casualties with and without PTSD, and interactions between whiplash and pain were investigated.

Statistical Analysis System software, version 9.3 for Windows (SAS Institute Inc., Cary, NC, USA) (proc genmod and proc glm) was used for all analyses.

#### Ethics and consent

The study protocol was submitted to and approved by the French Ministry of Research (CCTIRS: Advisory Committee on Information Processing in Material Research in the Field of Health) (CCTIRS Number 04.159). Data collection and analysis were approved by the national data protection authority (CNIL: CNIL Number 04–1417). Lastly, only patients (or their family) who gave written consent for follow-up were included in the cohort. At any time during the follow-up period, subjects were free to cease participation, and, in that case, to be totally withdrawn from the study files and analyses.

#### Availability of data

Data are available from the authors upon request (CNIL’s requirement).

## Results

### Representativeness of the study population

Respondents in the reference population (MAIS1, non-whiplash group) were, on average, older than non-respondents (34.0 ± 14.8 years vs. 30.1 ± 14.7 years at time of accident; *p* < 0.03). Their educational level was more often higher than school-leaving certificate (29.7 % vs. 16.7 %; *p* < 0.03). There was no significant difference in terms of gender, family situation, occupation at time of accident, type of road user, reason for travel or body region involved (head, face, neck, thorax, abdomen, spine, upper limbs, or lower limbs).

Mean age at time of accident was not significantly different between respondents and non-respondents in the whiplash group. However, respondents were more numerous in the 35–45 years age-group (25.7 % vs. 8.1 %) and less numerous in the 16–25 years age-group (27.5 % vs. 43.0 %) (chi^2^ test; *p* < 0.01). They were also more often in work at the time of the accident (71.9 % vs. 58.1 %; *p* < 0.03). There was no significant difference in terms of gender, family situation, educational level, type of road user, reason for travel or body region involved.

There was no significant difference between the two grades of whiplash (grades 1 and 2) for respondents and non-respondents on sociodemographic, accident-related or injury characteristics.

### Characteristics of the populations

Comparison between whiplash and non-whiplash casualties revealed several significant differences (Table 1). In particular, females were more numerous in the whiplash group; whiplash casualties were more frequently four-wheel motor-vehicle drivers, with accidents involving another motor vehicle, and with rear impact; subjective responsibility was less frequently reported in the whiplash group, while financial difficulties in the year before the accident were more frequent. By contrast, there were no significant differences between the whiplash and non-whiplash casualties in terms of psychological history or intention to lodge a complaint.

**Table 1:** Sociodemographic and accident-related characteristics at inclusion for whiplash victims and non-whiplash victims

		Non-whiplash victims	Whiplash victims	Chi^2^ test^*1*^	Whiplash victims by grade	
		*N* = 185	*N* = 167		Grade 1 = 63	Grade 2 = 104
		n (%)	n (%)	*P*-value	n (%)	n (%)
Gender				<0.0001		
	Female	72 (38.9)	106 (63.5)		40 (63.5)	66 (63.5)
	Male	113 (61.1)	61 (36.5)		23 (36.5)	38 (36.5)
Age at the accident				NS		
	16–24 years	65 (35.1)	46 (27.5)		20 (31.7)	26 (25.0)
	25–34 years	49 (26.5)	47 (28.1)		13 (20.6)	34 (32.7)
	35–44 years	30 (16.2)	43 (25.7)		16 (25.4)	27 (26.0)
	45–54 years	19 (10.3)	14 (8.4)		4 (6.3)	10 (9.6)
	≥55 years	22 (11.9)	17 (10.2)		10 (15.9)	7 (6.7)
Family situation				<0.05		
	Single	89 (48.1)	59 (35.3)		24 (38.1)	35 (33.7)
	In couple	79 (42.7)	86 (51.5)		32 (50.8)	54 (51.9)
	Separated, divorced, widowed	17 (9.2)	22 (13.2)		7 (11.1)	15 (14.4)
Educational level				NS		
	<school-leaving cert.	92 (49.7)	76 (45.5)		30 (47.6)	46 (44.2)
	school-leaving cert.	38 (20.5)	36 (21.6)		16 (25.4)	20 (19.2)
	>school-leaving cert.	55 (29.7)	55 (32.9)		17 (27.0)	38 (36.5)
Socio-occupational category				NS		
	Farming, trade	13 (7.0)	5 (3.0)		4 (6.3)	1 (1.0)
	Exec., sup. intellectual	22 (11.9)	23 (13.8)		8 (12.7)	15 (14.4)
	Intermediate	21 (11.4)	17 (10.2)		5 (7.9)	12 (11.5)
	Office worker	71 (38.4)	83 (49.7)		28 (44.4)	55 (52.9)
	Manual	20 (10.8)	11 (6.6)		3 (4.8)	8 (7.7)
	Student, housewife, other, no reply	38 (20.5)	28 (16.8)		15 (23.8)	13 (12.5)
Financial difficulties^4^ before accident				<0.05		
	No	147 (79.5)	116 (69.5)		45 (71.4)	71 (68.3)
	Yes	38 (20.5)	51 (30.5)		18 (28.6)	33 (31.7)
Reason for travel				NS		
	Journey to work/school	66 (35.7)	65 (38.9)		20 (31.7)	45 (43.3)
	Work purposes	6 (3.2)	6 (3.6)		4 (6.3)	2 (1.9)
	Other	113 (61.1)	96 (57.5)		39 (61.9)	57 (54.8)
Type of road-user				<0.0001		
	4-wheel motor	76 (41.1)	145 (86.8)		52 (82.5)	93 (89.4)
	Other	109 (58.9)	22 (13.2)		11 (17.5)	11 (10.6)
Place in vehicle				<0.01		
	Driver	128 (69.2)	134 (80.2)		53 (84.1)	81 (77.9)
	Front passenger	17 (9.2)	18 (10.8)		6 (9.5)	12 (11.5)
	Rear seat passenger	8 (4.3)	7 (4.2)		2 (3.2)	5 (4.8)
	Unknown passenger/no reply	32 (17.3)	8 (4.8)		2 (3.2)	6 (5.8)
Antagonist				<0.01		
	None	49 (26.5)	20 (12.0)		4 (6.3)	16 (15.4)
	Other (pedestrian, fixed obstacle…)	28 (15.1)	21 (12.6)		10 (15.9)	11 (10.6)
	Motor vehicle	108 (58.4)	126 (75.4)		49 (77.8)	77 (74.0)
Impact direction^5^				<0.0001		
	Frontal	33 (17.8)	35 (21.0)		12 (19.0)	23 (22.1)
	Rear	7 (3.8)	56 (33.5)		17 (27.0)	39 (37.5)
	Lateral Right	18 (9.7)	19 (11.4)		6 (9.5)	13 (12.5)
	Lateral Left	12 (6.5)	27 (16.2)		15 (23.8)	12 (11.5)
	Don’t know/no reply	115 (62.2)	30 (18.0)		13 (20.6)	17 (16.3)
Friend or family member involved^6^				NS^3^		
	No	142 (76.8)	115 (69.5)		45 (71.4)	70 (67.3)
	Yes	43 (23.2)	51 (30.5)		17 (27.0)	34 (32.7)
Intention to lodge a complaint				NS		
	No	111 (60.0)	99 (59.3)		34 (54.0)	65 (62.5)
	Yes	18 (9.7)	22 (13.2)		10 (15.9)	12 (11.5)
	Don’t know/no reply	56 30.3)	46 (27.5)		19 (30.2)	27 (26.0)
Subjective responsibility in accident				<0.0001		
	No	52 (28.1)	92 (55.1)		39 (61.9)	53 (51.0)
	Yes	48 (25.9)	34 (20.4)		12 (19.0)	22 (21.2)
	Don’t know/no reply	85 (45.9)	41 (24.6)		12 (19.0)	29 (27.9)
Psychological history^7^				NS		
	No	129 (69.7)	105 (62.9)		37 (58.7)	68 (65.4)
	Yes	56 (30.3)	62 (37.1)		26 (41.3)	36 (34.6)
PTSD in the 1st Year				0.05		
	No	162 (87.6)	139 (83.2)		52 (82.5)	87 (83.7)
	Yes	23 (12.4)	28 (16.8)		11 (17.5)	17 (16.3)

^1^The test compares the group of the whiplash victims (all together) with the non-whiplash group

^2^NS: non-significant

^3^Fisher’s exact test

^4^Combination of several variables: job loss, financial difficulties, failure over the 12 months before the accident

^5^The impact direction was known only for the 4-wheel motor users; other road users are classified “no apply”

^6^Some non-respondent subjects: total does not equal 100 %

^7^Combination of several variables: sleep disorder, consumption of antidepressants/anxiolytics, regular appointments with a psychologist over the 12 months before the accident

The reference population (*n* = 185) included 337 distinct lesions distributed between the 8 body regions (head, face, neck, thorax, abdomen, spine, upper limbs, and lower limbs): i.e., 1.8 minor lesions per casualty. The lower and upper limbs were the most frequently affected regions (35.0 and 23.1 %, respectively). The most frequent lesions were: lower-limb skin contusion (hematoma) (AIS code 810402; *n* = 32), knee contusion (AIS code 850802; *n* = 31), and shoulder contusion (AIS code 751010; *n* = 21).

In the year following the accident, 16.8 % of whiplash casualties presented PTSD (grade 1: 17.5 %; grade 2: 16.3 %; non-whiplash casualties: 12.4 %), with no significant difference between groups (Table 1). Grade 1 and 2 whiplash casualties were compared on all analyses; no significant differences were found.

### Consequences at 5 years

#### Pain and sequelae

Five years after the accident, whiplash casualties (in particular, grade 2) were twice as likely to report pain as non-whiplash casualties (40.7 % vs. 22.2 %) (Table 2). Whiplash casualties suffered from neck pain (grade 1, 22.2; grade 2, 33.7) and spine pain (other than cervical spine) (12.7 % and 20.2 %, respectively). Non-whiplash casualties complained of spine pain (7.0) and lower-limb pain (9.7 %). Only one in five of those reporting pain at 5 years had suffered from PTSD during the first year after the accident, whichever the group (53/68 whiplash and 33/41 non-whiplash casualties).

**Table 2:** Description of pain and sequelae for whiplash and non-whiplash victims, at 5 year post-road-accident

		Non-Whiplash victims	Whiplash victims	Chi^2^ test^1^	Whiplash victims by grade	
		N = 185	N = 197		Grade 1 = 63	Grade 2 = 104
		n (%)	n (%)	p-value	n (%)	n (%)
Presence of pain				<0.001		
	Yes	41 (22.2)	68 (40.7)		22 (34.9)	46 (44.2)
	No/don’t know/no reply	144 (77.8)	99 (69.3)		41 (65.1)	58 (55.8)
Pain by body region (present vs. absent)						
	Head	2 (1.1)	11 (6.6)	<0.01	2 (3.2)	9 (8.7)
	Face	2 (1.1)	0 -	NS^2-3^	0 (0.0)	0 (0.0)
	Neck	8 (4.3)	49 (29.3)	<0.0001	14 (22.2)	35 (33.7)
	Spine	13 (7.0)	29 (17.4)	<0.01	8 (12.7)	21 (20.2)
	Thorax	2 (1.1)	2 (1.2)	NS^3^	1 (1.6)	1 (1.0)
	Abdomen	0 (0.0)	2 (1.2)	NS^3^	1 (1.6)	1 (1.0)
	Upper limbs	9 (4.9)	12 (7.2)	NS	5 (7.9)	7 (6.7)
	Lower limbs	18 (9.7)	7 (4.2)	<0.05	4 (6.3)	3 (2.9)
Sequelae^4^ of injuries related to the accident (Yes vs. No/don’t know/no reply)						
	Esthetic (broken nose, scar…)	41 (22.2)	13 (7.8)	<0.001	7 (11.1)	6 (5.8)
	Psychic (feeling bad)	32 (17.3)	42 (25.1)	NS	13 (20.6)	29 (27.9)
	Psychocognitive (concentration or memory trouble)	8 (4.3)	18 (10.8)	NS	5 (7.9)	10 (9.6)
	Headache	18 (9.7)	45 (26.9)	<0.0001	15 (23.8)	30 (28.8)
	Limbs (joint stiffness, lameness, muscle weakness…)	18 (9.7)	18 (10.8)	NS	6 (9.5)	12 (11.5)
	Spine (joint stiffness, frailty…)	16 (8.6)	31 (18.6)	<0.01	8 (12.7)	23 (22.1)
	Neurological (paresthesia…)	5 (2.7)	8 (4.8)	NS	1 (1.6)	7 (6.7)
	Sensory disorders (anosmia, …)	5 (2.7)	9 (5.4)	NS	3 (4.8)	6 (5.8)
	Internal organs (dysfunction)	1 (0.5)	8 (4.8)	<0.02^3^	4 (6.3)	4 (3.8)
	Other	3 (1.6)	0 -	NS^3^	0 (0.0)	0 (0.0)

^1^The test compares the group of whiplash victims (all together) with the non-whiplash group

^2^NS: non-significant

^3^Fisher’s exact test

^4^other than pain

One in four whiplash casualty suffered from headache related to the accident (grades 1 and 2, 26.9 %; 10 % for non-whiplash casualties; *p* < 0.0001). Psychological sequelae were frequent in all three groups (grade 1, 20.6 %; grade 2, 27.9 %; non-whiplash, 17.3 %; non-significant difference).

There was no difference in terms of the impact of the accident on occupation or social and familial relations between whiplash and non-whiplash casualties (data not shown).

#### Quality of life – comparison between data at 1 and 5 years after the accident

In the whole group of respondents to the 5-year follow-up, one in four casualties reported less than good QoL. There was no significant difference between whiplash and non-whiplash casualties overall; however, grade-2 whiplash casualties were more dissatisfied with their health (39.4 %; *p* < 0.05) than non-whiplash (24.3 %) or grade-1 whiplash casualties (27.0 %); whiplash casualties’ mean scores were lower in the physical (grade 2, 75.2; grade 1, 76.5; vs. non-whiplash, 81.1; *p* < 0.01) and psychological domains (respectively: 67.6, 67.1, and 71.2; *p* < 0.05).

Restricting analysis to respondents at both follow-up steps (1 and 5 years), results were fairly comparable for the non-whiplash and whiplash groups (Table 3). Global QoL improved for both whiplash and non-whiplash casualties; but, considering the two whiplash groups separately, improvement in grade 2 was much less. In terms of overall health at 5 years, improvement was less in the whiplash (grades 1 and 2) than the non-whiplash group; taking the two whiplash groups separately, however, grade-1 casualties showed similar improvement to non-whiplash casualties, whereas grade-2 casualties reported a status quo (non-significant decline) in health status (Table 3).

**Table 3:** Comparison of the evolution of quality of life between 1 and 5 year after the accident

			Non-whiplash (*N* = 155)	Whiplash grade 1 et 2 (*N* = 133)	Whiplash grade 1 (*N* = 50)	Whiplash grade 2 (*N* = 83)
			n (%)	n (%)	n (%)	n (%)
Global Items						
Good quality of life^1^		1 year	108 (69.7)	95 (71.4)	34 (68.0)	61 (73.5)
		5 years	127 (81.9)	104 (78.2)	41 (82.0)	63 (75.9)
	Improvement (%)^2^		+*12.2* ^b^	+*4.8*	+*14.0*	+*2.4*
Satisfactory health status^3^		1 year	108 (69.7)	88 (66.2)	34 (68.0)	54 (65.1)
		5 years	117 (75.5)	90 (67.7)	38 (76.0)	52 (62.7)
	Improvement (%)^2^		+*5.8*	+*1.5*	+*8.0*	−*2.4*
Domains						
			m SD^4^	m SD^4^	m SD^4^	m SD^4^
Physical		1 year	77.1 (15.5)	72.8 (19.1)	73.4 (20.9)	72.4 (18.0)
		5 years	81.5 ^c^ (13.9)	76.2 (17.9)	76.0 (17.6)	76.2 (18.2)
	Improvement^5^		+*4.4* ^c^	+*3.4*	+*2.6*	+*3.8*
Psychological		1 year	66.6 (15.7)	64.6 (16.3)	61.6 (15.8)	66.5 (16.4)
		5 years	71.1 (13.6)	69.0 (15.3)	67.2 (17.0)	70.1 (14.2)
	Improvement^5^		+*4.5* ^c^	+*4.6* ^b^	+*5.6*	+*3.7* ^a^
Social		1 year	73.2 (19.5)	71.6 (19.9)	68.9 (20.9)	73.3 (19.3)
		5 years	75.5 (17.1)	74.7 (15.0)	73.3 (17.4)	75.5 (13.4)
	Improvement^5^		+*2.3*	+*3.3*	+*3.4*	+*2.3*
Environmental		1 year	68.0 (16.5)	65.0 (16.3)	65.3 (17.7)	64.8 (15.5)
		5 years	71.1 (15.4)	69.9 (16.6)	71.7 (15.9)	68.8 (17.0)
	Improvement^5^		+*3.1* ^b^	+*4.9* ^*c*^	+*6.4* ^a^	+*4.0* ^a^

(non-whiplash, whiplash grade 1; whiplash grade 2) (restricted to subjects who responded at both one and 5 years; *N* = 288)

^a^ <0.05; ^b^ <0.01; ^c^ <0.001

^1^Grouping: good/very good vs. neither good nor bad/ bad/very bad/no reply

^2^McNemar’s test

^3^Grouping: satisfied/very satisfied neither satisfied nor dissatisfied/dissatisfied/ very dissatisfied/ no reply

^4^SD: standard deviation

^5^Student’s test for matched data

For non-whiplash casualties, scores in the physical, psychological and environmental domains were significantly higher (*p* < 0.001) at five years than one year (Table 3); for the grade-1 whiplash group, the improvement was significant for the psychological and environmental domains (*p* = 0.03), whereas there was no significant improvement for the grade-2 whiplash group. Scores in the psychological, social and environmental domains were quite similar at five years in the various groups, but the physical score remained lower in the whiplash group.

### Multivariate models: factors for impaired quality of life at 5 years after accident

#### Perception of overall quality of life and of overall health

After adjustment on age and gender, whiplash status (Table 4) did not appear as a predictive factor for poorer overall QoL, but was related to unsatisfactory overall health, especially for grade-2 whiplash [RR = 1.48; 95%CI = 1.05–2.08]); other factors (educational level, psychological history, intention to lodge a complaint and PTSD) seemed also to be associated with poorer overall QoL and unsatisfactory overall health. Interestingly, the results for the two whiplash groups were inversed at the 1-year follow-up: RR was higher in grade 1 than grade 2.

**Table 4:** Factors related to “not good quality of life” and “unsatisfactory health” at 5 years

	Not good overall quality of life				Unsatisfactory health			
	Adj RR	95 % CI	Adj RR	95 % CI	Adj RR	95 % CI	Adj RR	95 % CI
	Model 1		Model 2		Model 1			Model 2
Adjustment factors								
Age > =35 years	2.10	1.41–3.13	1.95	1.30–2.92	1.23	0.89–1.69	1.17	0.87–1.57
Gender: female	1.29	0.85–1.94	1.13	0.74–1.72	1.27	0.91–1.76	1.00	0.73–1.37
Other factors								
Education level								
<school-leaving cert	1.43	0.82–2.47	1.10	0.61–1.99	1.69	1.05–2.70	-	
>school-leaving cert.	0.62	0.31–1.24	0.56	0.28–1.11	1.12	0.65–1.93	-	
Intention to lodge a complaint	-		-		1.64	1.11–2.42	-	
Psychological history	1.63	1.11–2.38	1.69	1.16–2.46	1.61	1.18–2.20	1.76	1.30–2.37
PTSD	1.97	1.30–2.98	1.78	1.17–2.70	1.61	1.16–2.24	1.48	1.10–1.99
Factor of interest								
Whiplash grade1	0.87	0.51–1.51	0.80	0.47–1.36	0.91	0.57–1.46	0.83	0.53–1.30
Whiplash grade2	1.43	0.93–2.17	1.24	0.81–1.90	1.48	1.05 –2.08	1.19	0.85–1.65
Suffering from Pain at 5 years	-	-	1.96	1.27–3.03	-	-	2.75	1.98–3.83

(modified Poisson regression models), model 1 without pain in the model; model 2 with pain)

When pain at five years was entered in the two previous models, it emerged as an intermediate factor for whiplash status (RR for whiplash status shifted from 1.43 to 1.24 (i.e., >10 % for overall QoL) and from 1.48 to 1.19 (i.e., 20 % for overall health)). Pain was significantly associated with poorer overall QoL (RR = 1.96; 95 % CI = [1.27–3.03]) and unsatisfactory health (RR = 2.75; 95%CI = [1.98–3.83]). Intention to lodge a complaint and educational level ceased to be significant for “health status” outcome in the final model.

There was no interaction between pain and PTSD in the relation between whiplash and unsatisfactory health, in spite of higher relative risk for pain in the whiplash than in the non-whiplash group (Table 5).

**Table 5:** Analysis of interactions between pain and PTSD related to unsatisfactory health

		Whiplash			No whiplash			
		Low health status	Good health status	RR_(pain if_ _Whiplash)_	Low health status	Good health status	RR_(pain if no Whiplash)_	Adjusted RR
		n	n		n	n		
No PTSD	Pain	28	25	3.03 (1.79–5.12)	13	20	2.31 (1.31–4.08)	2.67 (1.82–3.93)
	No pain	15	71		22	107		
PTSD	Pain	12	3	3.47 (1.24–9.65)	6	2	2.81 (1.11–7.13)	3.09 (1.55–6.15)
	No pain	3	10		4	11		

#### Scores on the 4 domains of quality of life

Once adjusted for age and gender, presence of whiplash showed no relation to QoL in 3 domains (mental: β = − 1.82 for grade 1 and β = − 2.14 for grade 2; social: respectively β = −1.00 and β = −1.12; or environmental: respectively β = +1.99 and β = −2.06)], and a borderline relation in the physical domain (β = −1.46 for grade 1 and β = − 3.71 for grade 2; *p* = 0.06). Psychological history and PTSD were predictive factors for impaired QoL in all 4 domains. Intention to lodge a complaint immediately after the accident was predictive in the physical domain, while educational level was not related to the social domain score.

Integrating pain into the four previous predictive models, whiplash status was non-significant in all four domains. Pain was a major factor for diminished QoL score, mainly in the physical and environmental domains: the regression coefficient (β) characterizing presence of pain five years after the accident was −13.55 (standard error (SE) = 1.79) for the physical domain, −4.35 (SE = 1.70) for the psychological domain, −5.85 (SE = 1.92) for the social domain and −8.55 (SE = 1.79) for the environmental domain. Pain was a major intermediate factor between whiplash and QoL.

## Discussion

### Interpretation of results

Few studies have been published on the long-term quality of life of mildly injured road-accident casualties. The present study focused on minor injury (M.AIS = 1) and tried to establish whether those suffering from whiplash (Quebec grades 1 and 2) differed in QoL five years after the accident. A further objective was to explore risk factors for impaired QoL. Results showed that, five years after mild road-crash injury, a significant number of patients still showed deteriorated quality of life, whatever the initial lesion. Furthermore, grade-2 whiplash subjects were more often dissatisfied with their health in terms of persistence of pain, and this was not explained by psychological history or development of PTSD.

Pain, headache and psychological sequelae related to the accident were strongly present in whiplash casualties five years after the accident, who were twice as likely to report pain as non-whiplash casualties (whiplash, 40.7 %; non-whiplash, 22.2 %), whereas pain was not more frequent in the whiplash group at 1 year [23]. This high percentage of residual pain is in agreement with literature reports. For example, Mayou et al. [17] showed that whiplash casualties were more often in pain than other casualty groups three years after the accident (whiplash injury, 30 %; other soft-tissue injury, 15 %; bone injury, 25 %; and no injury, 17 %), although these figures are much lower those reported by Stålnacke (63 % residual pain in whiplash casualties five years after the accident) [33]. Whiplash casualties in the present study most often reported pain located in the neck (29.3 %; 49 out of 167), compared to less than 5 % of non-whiplash casualties; these figures are lower than in a Swedish study [21] (39.6 % of whiplash and 14.0 % of non-whiplash casualties suffering from neck pain seven years after rear-end collision).

While overall QoL was not related to whiplash status, satisfaction with overall health was negatively correlated with whiplash, which is consistent with the analysis by domain: the only domain which was affected by whiplash was the physical domain. This was true even when gender, age, educational level, baseline psychological problems and post-accident PTSD were controlled for. Rebbeck et al. [8] also found a large proportion of subjects with whiplash not satisfied with their health status: almost half had not recovered two years after injury. Likewise, a recent Lithuanian study showed that whiplash casualties had worse general health status than matched controls [34].

Furthermore, comparison between data at one and five years showed an improvement in QoL in all groups (with or without whiplash) but, in grade-2 whiplash, QoL remained poorer than in the non-whiplash group five years after the accident; indeed, the mean physical score for the grade-2 whiplash group decreased at five years. Schwerla et al. [14] showed a significant improvement in the physical and mental component summary (SF-36) between the beginning and end of osteopathic treatment in subjects suffering from whiplash injuries. Rebbeck et al. [8] also showed significant improvement over time (follow-up at three and six months and two years) for the physical score (SF-36).

Psychological factors are often put forward as explaining the chronification of whiplash injury [11, 35]. In agreement with Holm [36], we think that non-recovery after a mild accident may be multifactorial and that psychological factors can be a contributing cause of WAD: in the present study, previous psychological factors were related to QoL in whiplash casualties. Similarly, PTSD is often suggested to be a predictive factor for poor QoL in the first years following an accident [37]: for example, in the present study, 16.8 % of the whiplash group were suffering from PTSD at the one-year follow-up (12.4 % for non-whiplash casualties; non-significant difference). Similar results were reported by Mayou^17^ at 3 years, whiplash casualties tending to show a higher rate of PTSD (17 %) than other groups (non-significant difference). In the present study, PTSD was a major predictor of lower scores for all three domains (mental, social, environmental), whereas whiplash status was not; but, while PTSD was a major factor for the physical domain, whiplash remained a predictive factor after adjustment on PTSD.

The role of compensation has often been raised [11, 15, 38–41]. Sterling et al., however, found no association between lodging a claim and the persistence of moderate/severe symptoms for twelve months in whiplash casualties [42]. Several points should be noted: the present results were adjusted for the intention to lodge a complaint, and there was no correlation between PTSD and intention to lodge a complaint. Furthermore, in France, the financial costs of care are covered by national health insurance for everybody, and only a small fraction of the mildly injured lodge a compensation claim against a private insurance company; furthermore, lodging a complaint with the court is not mandatory for a compensation claim to be lodged against an insurance company. Interestingly, in our models, in spite of adjustment on these various factors, the relation between whiplash and lower satisfaction with overall health remained statistically significant. The difference in rate of pain after whiplash between the present study and many others may be explained by the general frequency of compensation claims in the various countries; but other factors may also interfere, such as true neurophysiological disturbance.

In fact, in the causal chain between the accident and lower satisfaction with health-related quality of life, the main question is the exact role of pain in the relation between whiplash and impaired long-term QoL. It is suggested that pain is strongly correlated with severe PTSD [43], but in some traumas this relation is not so clear [44]. Like Egloff et al., comparing psychological conditions in patients with chronic pain disorder with and without non-dermatomal somatosensory deficit [45], we found no difference in baseline psychological condition between study groups. Analyzing overall health in whiplash and non-whiplash casualties found no interaction between PTSD and pain.

Considering that the only significant difference between the two whiplash groups concerned the circumstances of the accident, and notably that psychological conditions were similar, the lack of improvement in the grade-2 group, in marked contrast to the grade-1 group, which greatly improved, can only be explained by pain, possibly neuropathic, related to a more severe initial neck injury; this could explain the perception of poorer overall health in the grade-2 group, whereas the other domains were not related to whiplash. This point needs to be further investigated in future analyses.

### Strengths and limitations of the study

Few studies deal with the long-term consequences of mild injury. The present study was based on the ESPARR cohort, which prospectively follows up a population of 1,168 road-accident casualties for 5 years. The ESPARR cohort is not specific to whiplash injury, so it can be expected that there is no selection bias: all patients managed in a public or private-sector hospital in a well-defined geographical area are included, whatever the severity and type of road accident [26]. The present analysis focused on the mildly injured (MAIS-1) members of the cohort, which explains the relatively small number of whiplash casualties. On the one hand, as the cohort does not focus on whiplash, no specific whiplash scale analysis, such as the Neck Disability Index (NDI) [46] or Neck Pain and Disability Scale (NPDS) [47], was used; furthermore, the questions on pain were not specific to any particular body region (e.g., neck), and subjects were free to specify any body regions affected by pain, thus enabling comparison between the two groups of mild injury without specifically focusing on neck pain. We thereby avoided introducing an information bias, whereas numerous whiplash studies analyzed only cohorts of whiplash casualties claiming compensation [48] or under treatment for chronic pain [49]. The present pain assessment was based on subjective response; this precluded investigating the existence of a neuropathic component as proposed by Sterling and Pedler [19]. On the other hand, as discussed by Carlesso et al. [50], not using a specific neck-pain questionnaire may have hidden some disabilities specifically related to whiplash; finally, Skevington et al. [51] pointed out that the use of WHOQoL-Bref may lead to poor detection of subtle differences, notably in the social domain. WHOQOL was built up from a long process of work, discussion and validation between specialists from a number of countries; in particular, the mental facet was framed by the definitions given by the DSM-IV [52].

At five years, information was obtained through a self-administered questionnaire for all patients, and very few items were not answered; it is unlikely that bias could have been introduced in the analyses between the various groups, but it is possible.

Another point to be discussed is the possibility of an information bias related to non-respondents: the rate of non-response was quite similar in the whiplash and non-whiplash groups, and it is unlikely that this could have introduced any bias in the analysis. It seems that subjects who responded only to the five-year follow-up had slightly lower health status than those who responded to both follow-ups; but analysis restricted to the latter did not change the pattern of results. The same percentage of subjects participated in both follow-ups (one and five years) in the two groups of whiplash casualties (grade 1: 79.3 % and grade 2: 79.8 %), so the difference observed between the two grades was unrelated to response bias.

## Conclusion

Deteriorated quality of life in the mental, social and environmental domains was mainly related to psychological and socioeconomic factors for both whiplash casualties and other mildly injured road-accident casualties. However, unsatisfactory health at five years, with deteriorated quality of life in the physical domain, was observed specifically in the whiplash group, pain playing a predominant intermediate role, which was not found at the one-year follow-up. Grade-2 whiplash casualties were particularly affected. The hypothesis of neuropathic pain might usefully be further explored.

## Abbreviations

AIS: Abbreviated Injury Scale
95%CI: 95 % confidence interval
DSM-IV: Diagnostic and Statistical Manual of Mental Disorders, 4th Edition
ESPARR: Etude et Suivi d’une Population d’Accidentés de la Route dans le Rhône (Follow-up study of road-crash casualties in the Rhône administrative area)
MAIS: Maximum abbreviated injury scale
PTSD: Post-traumatic stress disorder
QoL: Quality of life
RR: Relative risk
WAD: Whiplash-associated disorder

## Footnotes

1 In France, medical costs are payed by the national health insurance scheme; financial compensation is obtained through the driver’s compulsory insurance. Lodging a complaint is essentially a way to obtain adjudication about responsibility, rather than financial compensation.
